# Aqueous Rechargeable Metal‐Ion Batteries Working at Subzero Temperatures

**DOI:** 10.1002/advs.202002590

**Published:** 2020-11-23

**Authors:** Yuwei Zhao, Ze Chen, Funian Mo, Donghong Wang, Ying Guo, Zhuoxin Liu, Xinliang Li, Qing Li, Guojin Liang, Chunyi Zhi

**Affiliations:** ^1^ Department of Materials Science and Engineering City University of Hong Kong Hong Kong 999077 China; ^2^ College of Materials Science and Engineering Shenzhen University Shenzhen 518060 China; ^3^ Centre for Functional Photonics City University of Hong Kong Kowloon 999077 Hong Kong

**Keywords:** aqueous batteries, low‐temperature batteries, low‐temperature energy storage, metal‐ion batteries, subzero temperature batteries

## Abstract

Aqueous rechargeable metal‐ion batteries (ARMBs) represent one of the current research frontiers due to their low cost, high safety, and other unique features. Evolving to a practically useful device, the ARMBs must be adaptable to various ambient, especially the cold weather. While much effort has been made on organic electrolyte batteries operating at low temperatures, the study on low‐temperature ARMBs is still in its infancy. The challenge mainly comes from water freezing at subzero temperatures, resulting in dramatically retarded kinetics. Here, the freezing behavior of water and its effects on subzero performances of ARMBs are first discussed. Then all strategies used to enhance subzero temperature performances of ARMBs by associating them with battery kinetics are summarized. The subzero temperature performances of ARMBs and organic electrolyte batteries are compared. The final section presents potential directions for further improvements and future perspectives of this thriving field.

## Graphical abstract

Based on the freezing behavior of water and its effects on subzero performances of aqueous rechargeable metal‐ion batteries (ARMBs), strategies used to enhance subzero performance of ARMBs by associating them with battery kinetics are summarized. The subzero performance of ARMBs and organic electrolyte batteries are compared. Future perspectives of this thriving field are presented.

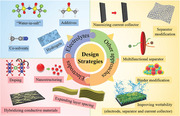

## 1 Introduction

Rechargeable batteries are used extensively in electrical energy storage (EES) for sustainable economic and social development.^[^ ^1^ ^]^ As the demand for energy storage continues to soar and more and more application scenarios emerge, batteries are urged to function in extreme weather. Nearly all types of batteries are less efficient in cold conditions, that is, battery life, capacity, and rate capability are all deteriorated at low temperature.^[^ ^2^ ^]^ In this regard, extending the low‐temperature ranges of batteries is significant in the performance advancements for EES and practical development such as operations of aerial vehicles, cybernators, and submarines. Currently, batteries based on organic electrolytes dominate the market, correspondingly, there are plenty of researches on the low‐temperature batteries with organic electrolyte.^[^ ^3^ ^]^

Aqueous rechargeable metal‐ion batteries (ARMBs) with the characteristics of high safety, low cost, easy‐to‐assemble, high ionic conductivity, and environmentally benign have been viewed as promising alternatives for some specific application scenarios, such as grid‐scale energy storage.^[^ ^4^ ^]^ Compared with their counterparts based on organic electrolytes, ARMBs use water as the electrolyte solvent, bearing following virtues: i) flame retardancy resulted from aqueous electrolytes, ii) easy to scale up on account of maneuverability in the air, iii) potentially better round‐trip efficiency ascribed to the higher ionic conductivity of aqueous electrolytes, iv) relatively low cost of water as solvents, and v) environment amity.^[^ ^5^ ^]^

Unfortunately, with water freezing, ARMBs naturally suffer from severe performance degradation at subzero temperatures, which results from deterioration of both electrolytes and electrode materials. The main mechanism for poor battery performance of ARMBs at subzero temperature are as follows: i) declined electronic conductivity and slower ionic diffusion of the electrode materials; ii) lower ionic conductivity of the electrolyte; and iii) slower charge transfer kinetics due to the decrease in the chemical reaction rate. Reasonably, strategies currently developed to improve the low‐temperature performance of ARMBs mainly rely on improving electron/ion and reaction kinetics of electrolytes, electrodes, and other support components, including separators, binders, and current collectors.

No review article has been published so far focusing on detailed analysis and systematic summary of progress on ARMBs working at subzero temperature. It is gripping and necessary to review this research frontier to examine the latest progress and potential guidance to improve the low‐temperature performance of ARMBs. The advances of innovative electrolytes, electrochemical properties of electrode materials, and the function of other supporting components of low‐temperature ARMBs are thoroughly discussed in the following sections. The optimization of battery performance under cold conditions with the inherent characteristics of these materials provides a favorable prospect for the grid‐scale applications of ARMBs.

## 2 Water Solution at Subzero Temperature

Water solutions are generally considered to be excellent electrolytic conductors as a result of the unique dielectric and fluid properties of water. Physical and chemical changes of water occurring at subzero temperatures induce multiple challenges to the low‐temperature operation of ARMBs. Freezing of aqueous electrolyte, inefficient ionic conductivity, and insufficient wettability at low temperatures are deemed to be the main reason resulting in battery performance degradation.^[^ ^2^, ^6^ ^]^

### 2.1 Water Freezing

**Figure 1:**
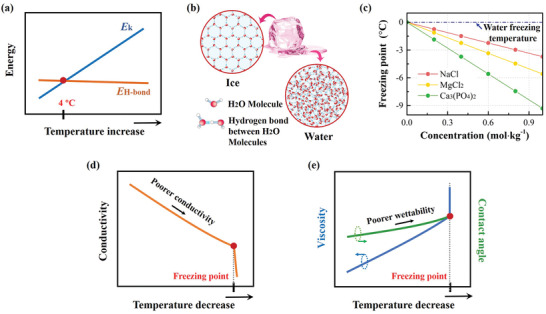
Schematics of the water solution at low temperature. a) Temperature‐dependent energy in pure water where *E*_k_ and *E*_H‐bond_ refer to the kinetic energy and the energy of the hydrogen bonds of water molecules, respectively. b) The crystal structures of water and ice with hydrogen bonds inside. c) The concentration‐dependent freezing point of water and three salt solutions. d) Temperature‐dependent conductivity of the water solution of highly concentrated salt. e) Temperature‐dependent viscosity and contact angle of the water solution.

Considering the great influence of water freezing to properties of aqueous electrolytes, we first elaborate on the mechanism of phase transition of water at subzero temperatures. Hydrogen bonding (O:H—O) is the basic structure and energy storage unit of water. Hydrogen atoms are shared in hydrogen bonds. Hydrogen bonds form an extended network between trillions of molecules and these bonds keep breaking and recombining. When the water cools down above 4 °C, the kinetic energy (*E*_k_) of the water molecules shortens the duration of the interactions with each other causing hydrogen bonds to form and break more quickly (**Figure** **1**a). When the temperature is below 4 °C, *E*_k_ of the water molecules starts to fall below the energy of the hydrogen bonds (*E*_H‐bond_) resulting in that hydrogen bonds form at much higher frequencies than they break. Finally, solid water—ice—is formed (Figure 1b).^[^ ^7^ ^]^

Therefore, it is a general approach to inhibit the formation of crystal lattices of ice at subzero temperature by means of endowing ions in the electrolyte form stronger hydrogen bonds with water molecules in competition with hydrogen bonds in water.^[^ ^8^ ^]^ The concept is widely adopted in developing electrolytes for ARMBs working at subzero temperatures. Employment of highly concentrated (or saturated) solutions of salt in water can effectively reduce the freezing point of water dozens of degrees.^[^ ^9^ ^]^ In a simple description, the drop of freezing point can be expressed by the equation

(1) $$\begin{array}{c} \Delta \;\;T_{f}=T_{\mathrm{water}}\;-\;T_{\mathrm{soln}}=K_{f}\;\cdot m \end{array}$$

where Δ*T*_f_ is changed in freezing point, *T*_water_ is the thermodynamic freezing point of water (0 °C), *T*_soln_ is the freezing point of the solution, *K*_f_ is freezing point depression constant which is different for each solvent (*K*_f_ = 1.86 °C kg mol for water), and *m* is the molality of solution (mol kg^−1^). For instance, the freezing point for 1 m NaCl, MgCl_2_, and Ca_3_(PO_4_)_2_ solutions are calculated to be −3.7, −5.6, and −9.3 °C (Figure 1c).^[^ ^10^ ^]^ The calculation process for NaCl (note that there are 2 m ions from Na^+^ and Cl^−^ ions) is as follows

(2) $$\begin{array}{ccc} \Delta \;\;T_{f} & = & \left(\frac{1.86{\;}^{\circ }C\;\mathrm{kg}\;H_{2}O}{\mathrm{mol}\;\mathrm{particles}}\right)\;\cdot \left(\frac{1\;\mathrm{mol}\;\mathrm{NaCl}}{1\;\mathrm{kg}\;H_{2}O}\right)\\ & & \cdot \;\left(\frac{2\;\mathrm{mol}\;\mathrm{particles}\;}{1\;\mathrm{mol}\;\mathrm{NaCl}}\right)=\;3.7{\;}^{\circ }C \end{array}$$

(3) $$\begin{array}{c} T_{\mathrm{soln}}=\;0{\;}^{\circ }C-3.7{\;}^{\circ }C\;=\;-3.7{\;}^{\circ }C \end{array}$$

### 2.2 Ionic Conductivity and Wettability Property

In a dilute salt solution, the decrease of temperature decelerates the movement of the charger carriers in a solution, but the conductivity may remain unaffected due to the limited number of charger carriers. However, in a highly concentrated salt solution, the conductivity may remarkably decrease with the declined temperature (Figure 1d).^[^ ^11^ ^]^ Moreover, the viscosity of electrolyte increases rapidly at low temperature (Figure 1e), which has adverse effects on ion migration and electrode infiltration, eventually sacrificing the rate performance of the battery.^[^ ^12^ ^]^ Another factor that may affect low‐temperature battery performance is that the declined temperature may deteriorate the wettability of solutions, which is manifested by the changed contact angle (Figure 1e). The contact angle will slightly increase with a declined temperature due to the increase in hydrogen bonds and surface tension.^[^ ^13^ ^]^

## 3 Strategies to Maintain the Performance of ARMBs at Subzero Temperature

**Figure 2:**
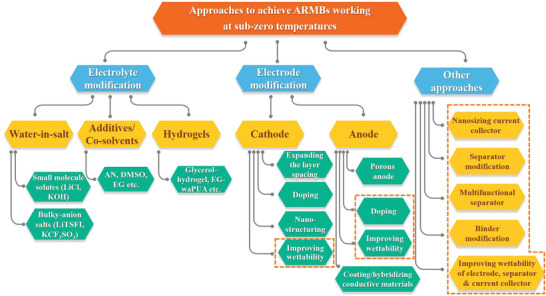
Summary of design strategies to improve the subzero‐temperature performance of ARMBs. Approaches circled by a dotted line are believed to be effective for ARMBs but have not been demonstrated. Some of them have been adopted for organic electrolyte batteries.

In this section, the developed strategies toward maintaining the electrochemical performance of ARMBs at subzero temperature will be introduced, which is summed up in **Figure** **2**. These strategies consist of three main approaches: electrolyte optimization, electrode design, and other inactive components of engineering. It should be mentioned that some approaches are believed to be effective and some of them have been adopted for organic battery systems, but so far, they have not been demonstrated in ARMBs. These approaches are also summarized and circled in dotted lines in Figure 2.

**Figure 3:**
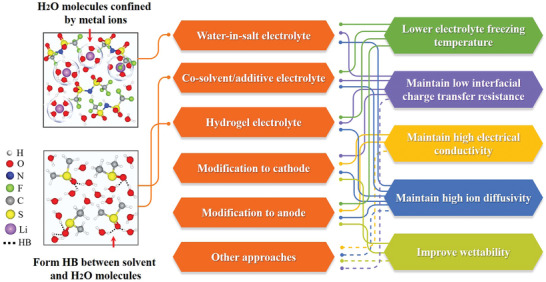
Summary of design mechanisms of these strategies to improve the subzero‐temperature performance of ARMBs. The mechanism diagrams at the top left and at the bottom left take LiTFSI “water‐in‐salt” electrolyte (the red arrow refers to Li^+^‐4(H_2_O) inorganic phase) and DMSO additive (the red arrow refers to HB formed between DMSO‐2(H_2_O)) as examples, respectively. HB here stands for hydrogen bond.

Corresponding to the degradation of components in an ARMB at subzero temperature, these approaches mainly take advantage of following mechanisms to achieve capacity retention at decreased temperature: lowering electrolyte freezing temperature, maintaining low interfacial charge transfer resistance, maintaining the high electrical conductivity of the electrode, maintaining high ion diffusivity of electrode and electrolyte, as well as improving interfacial wettability between electrode and electrolyte. We summarize the approaches and the corresponding mechanisms in **Figure** **3**. It should be noted that in all cases, one approach may benefit from a couple of mechanisms simultaneously. For example, an antifreeze hydrogel electrolyte can achieve lower electrolyte freezing temperature, meanwhile, the ion diffusivity can be maintained high and interfacial charge transfer resistance can be kept low.

### 3.1 Optimizing Aqueous Electrolytes

Electrolyte, as the ionic conductor, has a profound importance on the overall performance of ARMBs. In the following subsections, we discuss how to manipulate electrolytes to enhance the subzero‐temperature performance of ARMBs. This pertains to optimizing solvent composition, developing alternative electrolytes, employing multifunction additives and antifreeze hydrogels. These approaches have been proved to be feasible and effective.

#### 3.1.1 “Water‐in‐Salt” Electrolyte

Highly concentrated aqueous electrolytes are quite advantageous to endow ARMBs to work at subzero temperatures. Currently, they have been applied in aqueous lithium‐ion batteries (ALIBs), aqueous zinc‐ion batteries (AZIBs), aqueous potassium‐ion batteries (AKIBs), and metal‐air batteries. The useful solutes in different systems include LiCl, KOH, and bulky anion salts such as lithium bis(trifluoromethane sulfonyl) imide (LiTFSI) and its derivatives, as well as zinc trifluoromethanesulfonate (Zn(CF_3_SO_3_)_2_) and KCF_3_SO_3_.

LiCl saturated solution is very effective to maintain performances of ALIBs at subzero temperatures down to −45 °C. Employing a LiCoO_2_ (LCO) cathode, capacity retention of 72% can be obtained at −40 °C. This is even better than its organic electrolyte counterparts.^[^ ^10^ ^]^ Analogously, metal‐air batteries commonly prefer a slightly higher concentration aqueous alkaline electrolyte (30–40% KOH) for its subzero temperature applications (down to −30 °C).^[^ ^14^ ^]^ Bulky anion salts are also widely used for ARMBs working at subzero temperatures. The water solution of 21 m (mol kg^−1^) LiTFSI and its derivatives possess high electrochemical stability resulting from the ultralow‐concentration water molecules, almost all of which are strongly coordinated with Li^+^ and constrained in anion‐containing Li^+^ solvation sheaths.^[^ ^15^ ^]^ The Li^+^ solvation sheaths lead to the formation of dense interphase on the surface of the anode. With all‐NASICON electrode materials (Li_3_V_2_(PO_4_)_3_/LiTi_2_(PO_4_)_3_, LVP/LTP) and low freezing point of the 21 m LiTFSI aqueous electrolyte, an ALIB can deliver a superior reversible capacity of 111 mAh g^−1^ at −20 °C, which is very close to the 120 mAh g^−1^ at 25 °C.^[^ ^16^ ^]^ LiTFSI is also used as a concentration enhancer, which contributes to the stability of the electrolyte. A typical system is an electrolyte of 1 m Zn(CF_3_SO_3_)_2_ + 21 m LiTFSI, which is used to achieve an impressive electrochemical performance of an AZIB at −15 °C (60 mAh g^−1^).^[^ ^17^ ^]^ For AKIBs, 22 m KCF_3_SO_3_ water‐in‐salt electrolyte was demonstrated to be effective.^[^ ^18^ ^]^ At down to −20 °C, the battery retains 76% of its capacity (8.4 mAh). The CE of the pouch cell increases to near 99.9% (0.1C) at −20 °C owing to the effect of lower temperature on the fewer side reactions. Recently, novel water‐in‐salt electrolytes based on asymmetric anions have been developed to prevent crystallization at subzero temperatures. Asymmetric (fluorosulfonyl)(trifluoromethanesulfonyl)imide (FTFSI) and (pentafluoroethanesulfonyl)(trifluoromethane‐sulfonyl)imide (PTFSI) anions hinder the formation of long‐range order of high‐concentration electrolytes effectively suppressing crystallization.^[^ ^19^, ^20^ ^]^ By further mixing NaFTFSI with other anion salts (NaFSI), the liquid state of water‐in‐salt electrolytes could be extended to −14 °C.^[^ ^20^ ^]^

In terms of the mechanism, the freezing point of the “water‐in‐salt” electrolyte can be remarkably lowered from the perspective of thermodynamics. By choosing appropriate salts, the ionic conductivity of the “water‐in‐salt” electrolyte can also be decently kept at subzero temperature although a lower ionic conductivity is not avoidable. Besides, the formation of anodic‐derived stable and conductive solid‐electrolyte interphase (SEI) is beneficial for accelerating the phase transfer of metal ions and decrease charge transfer resistance.^[^ ^21^ ^]^ Notably, for the LiCl, two to three orders of magnitude higher conductivity in aqueous electrolyte than its organic electrolytes counterparts were achieved.^[^ ^2^, ^22^ ^]^ These factors undoubtedly contribute to ARMBs at subzero temperatures. Moreover, “water‐in‐salt” electrolyte also brings the following merits to ARMBs: i) alleviating the electrode dissolution and metal corrosion, further improving the stability of active materials and electrolyte compared to dilute solutions;^[^ ^23^ ^]^ ii) expanding the potential window;^[^ ^24^ ^]^ iii) promoting electrode reactions;^[^ ^25^ ^]^ iv) increasing metal ion transference number,^[^ ^26^ ^]^ and v) suppressing flammability and vaporability.^[^ ^27^ ^]^ These features significantly benefit ARMBs’ operation in cold environments. On the other hand, it is still fundamentally challenging but technically critical to overcoming intrinsic limitations of the “water‐in‐salt” electrolytes in the future, including low conductivity, high viscosity as a result of the strong cation–anion ion coupling, salt precipitation as the temperature goes down, and high cost.

#### 3.1.2 Cosolvents or Additives

Compared to traditional SEI, aqueous SEI originates from the reduction of salt anions in aforementioned “water‐in‐salt” electrolytes, suffering from a severe “cathodic challenge.” This is ascribed to the repulsion of anions by negatively polarized surfaces of the anode.^[^ ^28^ ^]^ Therefore, how to broaden the cathodic limit of the aqueous electrolyte in a cost‐efficient manner is also very important to enhance performance ARMBs at subzero temperatures. In terms of thermodynamics, the liquid range of electrolytes can be widened and a decent ionic conductivity can be maintained by adding cosolvents or additives with low melting point and low viscosity.^[^ ^29^ ^]^ In recent years, “hybrid aqueous/nonaqueous electrolyte” denotes a new class of cosolvents preserves inherent advantages of both aqueous and nonaqueous solvents, potentially resolving the conflicts among cost, interfacial chemistry, safety, and low‐temperature performance.^[^ ^30^ ^]^ On the other hand, recent highlights on low‐temperature additives are mainly concentrated in the field of nonaqueous batteries, and additives applied in aqueous batteries are in its juvenile stage.^[^ ^31^ ^]^ The effectiveness of additives to expand the low‐temperature range of ARMBs largely relies on the ability to reduce the freezing point of the electrolyte by inhibiting the formation of regular hydrogen bonds in ice (Figure 3). It should be noted that the terms of “cosolvents” and “additives” are both widely used in research. The main difference lies in the dosage used (unfortunately not strictly defined in reported research) but they usually share very similar mechanisms.^[^ ^32^ ^]^ Therefore, we discuss both of them in the same section. For convenience, we employ “additive” to illustrate the following context. Common additives utilized in aqueous electrolyte include acetonitrile (AN), dimethyl sulfoxide (DMSO), and ethylene glycol (EG).

**Figure 4:**
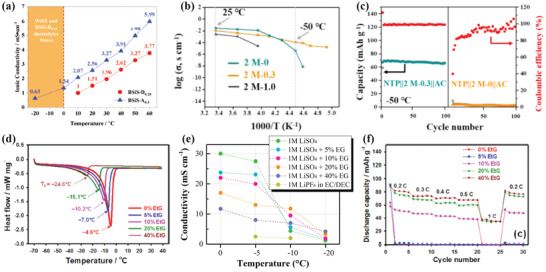
Improvement of low‐*T* performance by applying additives. a) Ionic conductivity of “bisolvent (water and DMC)‐in‐salt” BSiS‐D_0.28_ and “bisolvent (water and AN)‐in‐salt” BSiS‐A_0.5_ hybrid electrolytes at different temperatures (−20 to 60 °C). Reproduced with permission.^[^ ^68^ ^]^ Copyright 2019, Wiley‐VCH. b) Temperature‐dependent ionic conductivity investigation. c) Cycling performance of NaTi_2_(PO_4_)_3_@C (NTP)/2 m NaClO_4_ aqueous electrolyte with DMSO, *χ*_DMSO_ = 0.3 (2 m‐0.3)/activated carbon (AC) and NTP/2 m‐0/AC batteries at −50 °C. Reproduced with permission.^[^ ^6^ ^]^ Copyright 2018, Wiley‐VCH. d) DSC test on the aqueous electrolytes with EG at different mass fractions of 0, 5, 10, 20, and 40 wt%. e) Temperature‐dependent ionic conductivity of 1 m Li_2_SO_4_ electrolyte with different mass fractions of EG and the organic electrolyte of 1 m LiPF_6_ in EC/DEC (1:1, v/v). f) Rate capability of the LiFePO_4_ (LFP)/AC battery in 1 m Li_2_SO_4_ with different mass fractions of EG at −20 °C. Reproduced with permission.^[^ ^67^ ^]^ Copyright 2019, ACS.

AN possesses the merits of low freezing point (−48 °C), high dielectric constant (35.9), superior chemical and oxidative stability (>5 V vs Li^+^/Li), and high miscibility. Jointly use AN in a water‐in‐salt electrolyte, such as a hybrid electrolyte of “AN/21 m LiTFSI in the water” (AN‐WiSE) can provide remarkably improved ionic conductivity, reduced viscosity, and a lowered freezing point compared with the electrolyte of 21 m LiTFSI in water.^[^ ^33^ ^]^ This is because the spatial isolation ascribed from acetonitrile molecules and the maintained strong coordination between H_2_O molecules and Li^+^ lead to weakened electrostatic cation–anion attraction. Furthermore, the hybrid aqueous/nonaqueous and concentrated “AN and water (1:1 molar ratio)‐in‐salt” (BSiS‐A_0.5_) electrolyte exhibits evidently high activity of AN‐2Li^+^ solvation structure in electrochemical reduction. The prevailing species of Li^+^‐4(H_2_O) inorganic phase and fast exchange of AN organic phase are in favor of fast Li^+^ conduction, lower freezing point, as well as better wettability (**Figure** **4**a).^[^ ^10^, ^34^ ^]^ The assembled LiMn_2_O_4_/Li_4_Ti_5_O_12_ battery using BSiS‐A_0.5_ delivers a discharge capacity of 110 mAh g^−1^ (76% capacity retention) after 120 cycles at 0 °C, evidenced by much lower charge transfer resistance in BSiS‐A_0.5_ at 0 °C.

By employing DMSO as an additive, an electrolyte with freezing point lower than −130 °C and admirable ionic conductivity of 0.11 mS cm^−1^ at −50 °C was developed (Figure 4b).^[^ ^6^ ^]^ With the addition of DMSO, the hydrogen bonds between water molecules are greatly weakened and in contrast, the hydrogen bonds between water and DMSO molecules become much stronger.^[^ ^35^ ^]^ Also, 2 m‐0.3 (2 m NaClO_4_ aqueous electrolyte with DMSO, *χ*_DMSO_ = 0.3) state is fairly stable for forming DMSO–water hydrogen bonds and most of its molecules gather in the form of 2water–1DMSO, which prevents the mixture from generating ordered crystalline structure (ice) at low temperature and thus makes for lowering the freezing point.^[^ ^36^ ^]^ By using the 2 m‐0.3, NaTi_2_(PO_4_)_3_@C (NTP)/2 m‐0.3/activated carbon (AC) battery shows superior cycling performance at −50 °C (Figure 4c).

Among various antifreeze additives, the well‐known EG has many practical applications in electronics and automobiles. A perspective approach for the improved low‐temperature performance of an ALIB was developed by employing EG. With the increased amount of EG (0–40 wt%) in 1 m Li_2_SO_4_ electrolyte, the crystallization temperature decreases from −4.6 to −24.6 °C (Figure 4d). Furthermore, the best low‐temperature rate capability of the LiFePO_4_ (LFP)/AC battery is obtained in 1 m Li_2_SO_4_ with 40 wt% of EG at −20 °C due to the improved ionic mobility by the lower freezing point (Figure 4e,f). Similarly, a hybrid electrolyte of EG and ZnSO_4_ was applied in aqueous Zn//PANI‐V_2_O_5_ batteries.^[37]^ The hydrogen bonding between EG and H_2_O is significantly enhanced due to unique solvation interaction between EG and Zn^2+^, which endow the hybrid electrolyte with a low freezing point of −33 °C. Particularly, the battery retains 72% (130 mAh g^−1^) of its room‐temperature capacity at −20 °C.

In addition, blending additives in the electrolyte of Zn‐air batteries can also reduce the side reactions, such as hydrogen evolution reaction.^[^ ^38^ ^]^ The additive adsorbs on the active hydrogen evolution sites, reducing the solubility of Zn(OH)_4_^2−^ and further inhibiting the precipitation of ZnO.^[^ ^39^ ^]^ This approach improves the anode reaction efficiency and reduces the internal resistance, which is also conducive to the subzero‐temperature operation of Zn‐air batteries.

There is still plenty of room for exploring other additives with low melting point and low viscosity, such as diethyl carbonate (DEC), methylpropyl carbonate (MPC), methyl‐ethyl carbonate (EMC), and propylene carbonate (PC) to develop economic friendly low‐temperature ARMBs working at subzero temperature. Also, the introduction of organic additives in ARMBs is of great significance to construct SEI, which may effectively accommodate low‐temperature environments by preserving the merits of aqueous and nonaqueous solvents. It should be noted that usually very safe organic additives should be selected, that is, people do not want to scarify the unique safety nature of ARMBs to achieve their low‐temperature performances.

#### 3.1.3 Hydrogels

Except for adjusting the composition of the electrolyte, the development of hydrogels, a crosslinked meshwork of hydrophilic polymer chains dispersed in water, is also conducive to mitigating the dissolution issue of electrodes and alleviating deterioration of battery performance at subzero temperature. Hydrogels with nondrying and antifreezing (AF) properties have made remarkable progress in past years, which endows the application of hydrogels in low‐temperature ARMBs. Introducing cryoprotectants (CPAs) with low evaporation such as EG,^[^ ^40^ ^]^ glycerol,^[^ ^41^ ^]^ sorbitol, and oil^[^ ^42^ ^]^ into hydrogels is an effective method to prevent water loss and inhibit icing of water. It should be specially noted that, with antifreeze hydrogel electrolyte, flexible quasi‐solid‐state batteries working at subzero temperature can be fabricated benefiting from the well‐kept flexibility of the developed hydrogels at subzero temperature.

**Figure 5:**
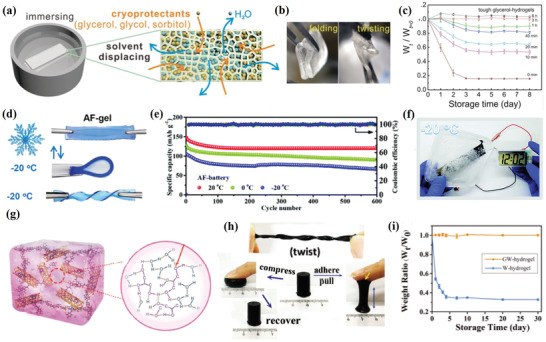
Improvement of low‐*T* performance by using hydrogels. a) Schematic of the preparation of tough organohydrogels by in situ replacing tough hydrogels with cryoprotectants (CPAs). b) Photographs of mechanical deformation of antifreezing (AF) glycerol hydrogels at −50 °C in folded and twisted states. c) Weight change of the tough glycerol hydrogels stored at 50% humidity and 20 °C. Reproduced with permission.^[^ ^43^ ^]^ Copyright 2018, Wiley‐VCH. d) Photographs of mechanical deformation of the AF‐gel at −20 °C in bent and twisted states. e) Cycling stability of the AF battery at 2.4 A g^−1^ and different temperatures. f) Photograph of quasi‐solid flexible AF batteries operated at −20 °C. Reproduced with permission.^[^ ^44^ ^]^ Copyright 2015, RSC. g) Schematic diagram for the glycerol‐water hydrogel (GW hydrogel) with polydopamine (PDA)‐modified carbon nanotubes (CNTs) well dispersed in acid (PAM‐*co*‐PAA) networks. h) Photographs of GW hydrogel after cooling at −20 °C for 24 h in twisted, compressed, and pulled states. i) Time‐dependent weight change of the GW hydrogel and water hydrogel (W hydrogel). *W*_0_ and *W*_t_ are the initial weight and real‐time weight at specific time, respectively. Reproduced with permission.^[^ ^41^ ^]^ Copyright 2017, Wiley‐VCH.

A straightforward, reliable, and versatile approach was developed by displacing solvent to fabricate antifreezing CPA‐based glycerol hydrogel with Ca‐alginate/polyacrylamide (PAAm) working at −50 °C (**Figure** **5**a–c).^[^ ^43^ ^]^ Moreover, an EG‐based waterborne anionic polyurethane acrylates (EG‐waPUA) hydrogel electrolyte was developed in a flexible Zn/MnO_2_ battery.^[^ ^44^ ^]^ The specific capacity of AF battery retains 74.54% over 600 cycles at −20 °C with CE approaching 100% (Figure 5d–f). The common link between aforementioned AF hydrogels is that the strong hydrogen bonds formed between glycerol (or EG) and water molecules prominently depress the freezing point and hold high conductivity at subzero temperature.^[^ ^45^ ^]^ Another class of hydrogels relies on the colligative property of ionic compounds such as calcium chloride (CaCl_2_) to inhibit the ice crystallization of the aqueous phase.^[^ ^46^ ^]^ In addition, as the interaction among terminal groups of polymer skeleton, electrolyte ions, and water has great influence on the AF performance of hydrogel electrolytes, introducing groups that can interact strongly with water into the hydrogel chain also reduces the freezing point of hydrogel electrolyte.^[^ ^47^ ^]^ By basifying the terminal group of the polyacrylic acid chains into a salt form (A‐PAA hydrogel) to obtain a stronger electrostatic attractive force, the freezing of water is greatly inhibited and thus the fabricated zinc‐air battery shows high capacity retention of 92.7% (691 mAh g^−1^) at −20 °C.^[^ ^48^ ^]^ Another polydopamine (PDA)‐modified carbon nanotubes grafted glycerol‐water hydrogel (GW hydrogel) was also proposed, which benefits from the covalent/noncovalent interactions in the glycerol‐water polymer chains and nano‐reinforcing carbon nanotubes synergistically.^[^ ^41^ ^]^ Viscous glycerol and catechol groups from PDA impart GW hydrogel with high tissue viscosity (Figure 5g–i). Remarkably, these AF hydrogels are mechanically flexible and can withstand a variety of large deformation (for instance, twisting, compressing, and pulling) even at subzero temperature. Thus, flexible ARMBs working at subzero temperatures were developed with both electrochemical performance and mechanical durability well kept.

It should be noted that the hydrogel strategy is usually used in combination with cosolvents, additives, or high‐concentrated salts to further broaden the low‐temperature range of electrolytes. For instance, the commonly reported hydrogel electrolyte containing highly concentrated salts enables batteries with antifreeze properties,^[^ ^49^ ^]^ high conductivity,^[^ ^17^, ^50^ ^]^ and excellent mechanical properties.

### 3.2 Electrode and Inactive Component

As the transfer centers for metal cations and electrons, the electrodes are also critical for ARMBs working at subzero temperatures. Generally, the low‐temperature performance of electrodes suffers from intrinsic sluggish kinetics ascribed to lower‐than‐usual electronic conductivity and ion diffusion capability. The electrochemical properties of electrodes are associated with the kinetic diffusion coefficient, the activation energy for diffusion, and the ability to decrease charge transfer resistance, which depends greatly on the structural characteristics of electrodes. With the strategies of expanding the layer spacing and increasing the specific area, the degradation of electrode performances at subzero temperatures can be suppressed effectively. Reports pertaining to the introduction of the structural water or radicals or metal ions and the fabrication of nanoporous structure are discussed in the ensuing sections.

On the other hand, while limited studies have been conducted on low‐temperature electrodes, there are many studies on strategies to improve the performance of the electrodes at room temperature. It is believed that these strategies will also be useful for the improvement of their subzero temperature performance due to the benefits of ion diffusion or electrode conductivity.

#### 3.2.1 Cathode

**Figure 6:**
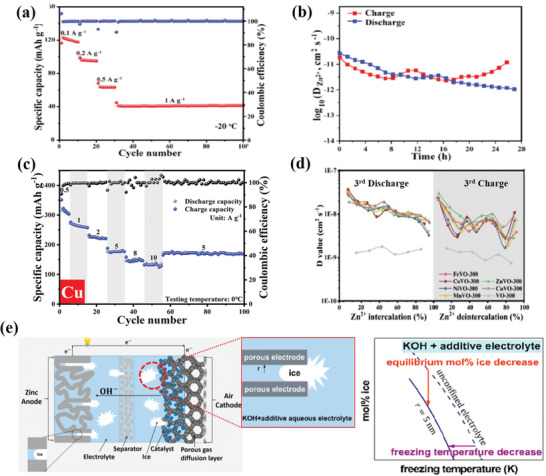
Improvement of low‐*T* performance by optimizing electrode materials. a) Rate capability at the current densities from 0.1 to 1 A g^−1^ followed by cycling of Zn//(NH_4_)_2_V_6_O_16_⋅1.5H_2_O (NVO) battery at 1 A g^−1^ and −20 °C. b) Zn^2+^ diffusion coefficient (*D*) in the second cycle of NVO. Reproduced with permission.^[^ ^51^ ^]^ Copyright 2019, Elsevier. c) Rate capability at the current densities from 0.5 to 10 A g^−1^ followed by cycling of Cu*_x_*V_2_O_5_·*n*H_2_O (annealed at 300 °C, CuVO‐300) at 5 A g^−1^ and 0 °C. d) *D* of Zn^2+^ in discharge/charge processes in the third cycle of transition metal ion‐preintercalated V_2_O_5_ (TVO)‐300 and V_2_O_5_ (VO)‐300. Reproduced with permission.^[^ ^53^ ^]^ Copyright 2019, Elsevier. e) Schematic illustration of zinc‐air battery configuration and the formation of ice at low temperatures. Reproduced with permission.^[^ ^56^ ^]^ Copyright 2018, ACS.

Vanadium materials are widely used as cathodes for ARZBs and their layered structure can be expanded to enhance electrochemical performance even at low temperatures.^[^ ^51^ ^]^ For example, inserted ammonium ions and crystal water expand the lattice spacing of V_3_O_8_ providing sufficient diffusion space for guest Zn^2+^. It delivers superior rate stability and maintains 120 mAh g^−1^ (32% capacity retention) at −20 °C benefiting from the enhanced kinetic Zn^2+^ transfer, even faster than the Li^+^ diffusion in LiV_3_O_8_ and LiFePO_4_ cathodes (**Figure** **6**a,b).^[^ ^51^, ^52^ ^]^ Another strategy of doping metal ions (Fe^2+^, Co^2+^, Ni^2+^, Mn^2+^, Zn^2+^, and Cu^2+^) in V_2_O_5_ (VO) cathode for vanadium‐based AZIB also afford improved ion diffusion ability, electronic conductivity, and structural stability during cycling at 0 °C, thus obtaining the excellent electrochemical performance of 80% capacity retention (Figure 6c,d).^[^ ^53^ ^]^ It should be noted that nanostructuring, and coating or hybridizing conductive materials are also beneficial for the improvement of subzero performance.^[^ ^54^ ^]^ An aqueous dual‐ion battery consisting of nano/microstructured Ni(OH)_2_ cathode and carbon‐coated NaTi_2_(PO_4_)_3_ anode displays high capacity retention of 85% after 10 000 cycles at −20 °C in 2 m NaClO_4_ electrolyte.^[^ ^55^ ^]^

While the research on low‐temperature cathodes for ARMBs are very limited, improving the wettability of cathodes are widely used approaches in low‐temperature organic electrolyte LIBs, which may inspire research on ARZBs at a subzero temperature in near future.^[^ ^2^ ^]^ In addition, it is imperative to explore some novel materials with high electronic and ionic conductivities at low temperatures, such as topological materials and defective semiconductor materials.

#### 3.2.2 Anode

Apart from the above progress on cathode materials, the anode is of vital importance on low‐temperature behavior for ARMBs as well. Adopting a nanoporous zinc anode was found effective to improve the low‐temperature performance of a zinc‐air battery (Figure 6e).^[^ ^56^ ^]^ The decrease in freezing point of the aqueous solution trapped in a pore can be represented as a function of pore radius (*r*) and concentration,^[^ ^57^ ^]^ and the function is a nonideal form of the Gibbs–Thomson equation as follows

(4) $$\begin{array}{c} T_{m,1}-T\;=\;\frac{2v_{1}^{S}\sigma^{\mathrm{SL}}T_{m,1}\cos\theta }{r\Delta H_{1}^{\mathrm{fus}}\left(1+\frac{RT_{m,1}\tilde{\pi }}{\Delta H_{1}^{\mathrm{fus}}}\right)}+\frac{R\tilde{\pi }T_{m,1}^{2}/\Delta H_{1}^{\mathrm{fus}}}{1+R\tilde{\pi }T_{m,1}/\Delta H_{1}^{\mathrm{fus}}} \end{array}$$

where *T*_m,1_ and *T* are the melting point of pure water at the bulk phase pressure and the freezing point of the aqueous solution, respectively. $v_{1}^{S}$ refers to the molar volume of pure water in the solid phase at *T*_m,1_ and liquid phase pressure (*P* ^L^). *R*, *σ* ^SL^, and *θ* are the universal gas constant, interfacial tension of the ice and solution, and the contact angle between the hole wall and the solid–liquid interface, respectively. $\Delta H_{1}^{\mathrm{fus}}$ and $\tilde{\pi }$ are the osmole fraction of the solution and the molar enthalpy of melting for pure water at *T*_m,1_ and *P* ^L^, respectively. The solution nonideality of multisolute solutions can be predicted from tabulated single‐solute properties using the Elliott and co‐workers form of the osmotic virial equation^[^ ^57^ ^]^

(5) $$\begin{array}{ccc} \tilde{\pi } & = & \sum_{i=2}^{z}k_{i}^{*}x_{i}\;+\sum_{i\;=\;2}^{z}\sum_{j\;=\;2}^{z}\left[\frac{\left(B_{ii}^{*}+B_{jj}^{*}\right)}{2}k_{i}^{*}x_{i}k_{j}^{*}x_{j}\right]+\;\sum_{i\;=\;2}^{z}\\ & & \sum_{j\;=\;2}^{z}\sum_{k\;=\;2}^{z}\left[{\left(C_{iii}^{*}C_{jjj}^{*}C_{kkk}^{*}\right)}^{1/3}k_{i}^{*}x_{i}k_{j}^{*}x_{j}k_{k}^{*}x_{k}\right]\;+\cdot \cdot \cdot \end{array}$$

where *i*, *j*, *k* = 2 and *z* refers to the *z*−1 solutes in the solution. *x_i_*, $B_{ii}^{*}$, and $C_{iii}^{*}$ are the mole fraction, the second and third mole‐fraction‐based osmotic virial coefficients of solute *i*, respectively. The second osmotic virial coefficient and the third osmotic virial coefficient refer to the interactions between two solute *i* molecules and interactions between three solute *i* molecules, respectively, etc. If solute *i* is thermodynamically ideal, then these coefficients are zero. For electrolyte solutes, solute concentration must be multiplied by $k_{i}^{*}$ (the mole‐fraction‐based dissociation constant of solute *i*). To be specific, as the temperature decreases, ice forms in the electrolyte. However, the formation or propagation of ice in the pores of the two electrodes requires lower temperatures than an unconfined electrolyte based on Equations (4) and (5). The calculation results show that the capillary pore with the diameter of 10 nm can reduce the local freezing point of the electrolyte by ≈10 °C. Thus, the performance of the electrolyte can be adjusted by designing the porous structure and reducing the particle size of the electrodes. However, a clear experimental demonstration of the effectiveness of this approach is not available yet. Similar to cathode research, studies on developing low‐temperature anode are also very limited, especially when the metal anode is employed. However, experience in improving the performance of anodes of organic electrolyte batteries at low temperatures is worthy of reference. For instance, the Li_3_V_2_(PO_4_)_3_/C (LVP/C) composite cathode exhibits reversible discharge capacity of 108.1 mAh g^−1^ (86.7% capacity retention) at −20 °C with traditional organic electrolyte benefiting from a modified electrode.^[^ ^58^ ^]^ Based on the above evidence, we suggest that low‐temperature performance for ARMBs can also be promoted by doping metal ions in electrodes.

#### 3.2.3 Inactive Component

Besides, the health of inactive components is also critical to maintaining low‐temperature battery operation. Although there are very limited reports on the temperature effect on electrochemically inactive components in ARMBs, some research has been carried out for organic electrolyte batteries and valuable experience can be learned. Approaches including utilizing multifunctional binders, modifying separators, and optimizing current collectors are helpful to sustain battery work at subzero temperature. Engineering separator with superior permeability,^[^ ^59^ ^]^ and endowing current collector with admirable contact area and conductivity are beneficial for reducing charge transfer resistance and promoting ions transfer.^[^ ^60^ ^]^ These are all successfully demonstrated in organic electrolyte batteries. Generally, it is believed that introducing nanoscale pores in the separator and current collector can also alter the freezing behavior of ARMBs based on the previously mentioned multisolute osmotic virial equation by Elliott and co‐workers.^[56b,^ ^60^ ^]^ These universal methods can inspire the design of inactive components in ARMBs working at subzero temperatures.

## 4 Performance Summary with an Emphasis on Comparison

Currently, the understanding on the mechanism of organic battery performance degradation at low temperatures is still limited, but technological challenges are partly blamed on the inherent kinetic restrictions analogous to that of ARMBs, such as charge transfer resistance, ionic conductivity, and melting point of electrolyte. Traditional organic electrolytes of LIBs have slower ionic transport at low temperatures leading to poor low‐temperature battery performances.^[^ ^61^ ^]^ Moreover, high desolvation penalties associated with strong Coulomb repulsion at the interface and high activation energies along with large interfacial charge transfer impedance in the organic electrolyte batteries impose restrictions on rate capability.^[^ ^62^ ^]^ Therefore, strategies to improve low‐temperature performance in organic electrolyte batteries mainly focus on improving electrode materials, electrolytes, and other support components to embellish the contact interfaces,^[^ ^32^ ^]^ and produce more stable and conductive SEI/cathode electrolyte interphase, which ultimately are helpful to facilitate the phase transfer of metal ions and reduce charge transfer resistance.^[^ ^21^, ^63^ ^]^

**Figure 7:**
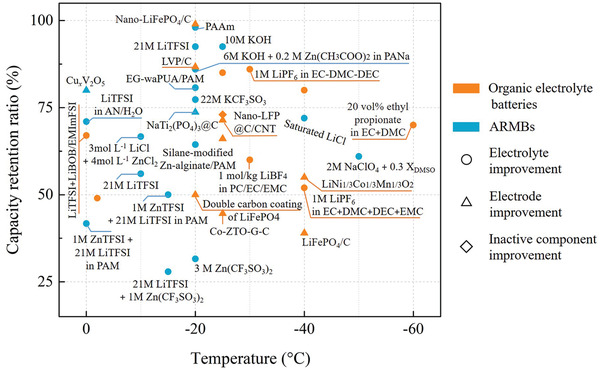
Relative capacity retention versus temperature for representative ARMBs,^[^ ^7^, ^14^, ^17^, ^33^, ^38^, ^39^, ^43^, ^51^, ^52^, ^53^, ^54^, ^55^, ^56^, ^57^ ^]^ and organic electrolyte batteries.^[^ ^54^, ^58^, ^60^, ^65^, ^66^ ^]^ Different shapes of the dots are used to represent different mechanisms and the notes for each dots are the key materials used to achieve the low‐temperature performances.

The reported temperature‐dependent capacity retention of representative ARMBs and organic electrolyte batteries is shown summarized in **Figure** **7**. The best record of 98% at present in ARMBs is realized in LiFePO_4_//2 mol L^−1^ ZnSO_4_ + 1 mol L^−1^ LiCl in PAAm hydrogel//Zn system at −20 °C,^[^ ^64^ ^]^ meriting from the combination strategies of high concentration of salt and hydrogel. The lowest temperature for the ARMBs system is observed at −50 °C (61% capacity retention), which is realized in the activated carbon//2 m NaClO_4_ + 0.3 X_DMSO_//NaTi_2_(PO_4_)_3_@C system, helped by the DMSO additive. Another decent performance is achieved in the LiCoO_2_//saturated LiCl//Li_0.75_CoO_2_ battery with 72% capacity retention at −40 °C. Also, the best record of 99% in organic systems is realized in nano‐LiFePO_4_/C//1 m LiPF_6_ in EC + DMC //Li system at −20 °C.^[^ ^65^ ^]^ It should be noted that many organic electrolyte batteries achieve decent low‐temperature performances at ≤−30 °C, while data of ARMBs are largely concentrated at temperature ≥−20 °C. This is related to the fact that organic systems naturally have lower freezing points than the aqueous counterpart. The detailed performance of representative subzero performances of ARMBs including ALIBs, ASIBs, AKIBs, AZIBs, and alkaline batteries are summarized in **Table** **1**.

**Table 1:** Summary of subzero‐temperature performances of representative ARMBs

Cathode	Anode	Electrolyte	*T* [°C]	Specific capacity [mAh g^−1^]	Capacity retention relative to RT capacity/RT capacity	Capacity retention/cycles (no. of cycles)	Rate capability [mAh g^−1^]
ALIBs							
LiCoO_2_^[^ ^10^ ^]^	Li_0.75_CoO_2_	Saturated LiCl	−40	65 at 0.2C	72% (90)	–	42 at 0.5C
Nano‐LiFePO_4_^[^ ^67^ ^]^	Activated carbon	1 m Li_2_SO_4_ + 40 wt% EG	−20	80 at 0.2C	–	65% (100)	35 at 1C
LiMn_2_O_4_^[^ ^68^ ^]^	Li_4_Ti_5_O_12_	LiTFSI in AN/H_2_O cosolvent	0	116 at 1C	71% (163)	95% (120)	–
LiFePO_4_^[^ ^69^ ^]^	Mo_6_S_8_	21 m LiTFSI	−10	23 at 0.2C	56% (41)	–	–
Li_3_V_2_(PO_4_)_3_^[^ ^16^ ^]^	LiTi_2_(PO_4_)_3_	21 m LiTFSI	−20	111 at 0.2C	92.5% (120)	–	66.7 at 6C
ASIBs							
Ni(OH)_2_^[^ ^55^ ^]^	NaTi_2_(PO_4_)_3_@C	2 m NaClO_4_	−20	70 at 10C	73.7% (95)	85% (10 000)	–
Activated carbon^[^ ^6^ ^]^	NaTi_2_(PO_4_)_3_@C	2 m NaClO_4_ + 0.3 *X*_DMSO_	−50	68 at 0.5C	61% (111)	94.3% (100)	46 at 10C
AKIBs							
KFeMnHCF‐3565^[^ ^18^ ^]^	PTCDI	22 m KCF_3_SO_3_	−20	8.5 mAh at 0.1C	77.3% (11 mAh)	–	9.1 mAh at 0.5C (−10 °C)
AZIBs							
*α*‐MnO_2_/CNT^[^ ^44^ ^]^	Zn	2 m ZnSO_4_ + 0.1 m MnSO_4_ in EG‐waPUA/PAM hydrogel	−20	226 at 0.2 A g^−1^	80.7% (243)	74.54% (600) at 2.4 A g^−1^	106 at 2.4 A g^−1^
LiMn_2_O_4_^[^ ^50^ ^]^	Zn	0.5 mol L^−1^ Li_2_SO_4_ + 0.2 mol L^−1^ ZnSO_4_ in silane‐modified Zn‐alginate/PAM organohydrogel	−20	60 at 0.3 A g^−1^	64.4% (90)	91.17% (100)	–
(NH_4_)_2_V_6_O_16_⋅ 1.5H_2_O^[^ ^51^ ^]^	Zn	3 m Zn(CF_3_SO_3_)_2_	−20	120 at 0.1 A g^−1^	31.58% (380)	98% (100) at 1 A g^−1^	41 at 1 A g^−1^
CuVO^[^ ^53^ ^]^	Zn	2 m ZnSO_4_	0	320 at 0.5 A g^−1^	80% (400)	–	134 at 10 A g^−1^
LiFePO_4_^[^ ^64^ ^]^	Zn	2 mol L^−1^ ZnSO_4_ + 1 mol L^−1^ LiCl in PAAm hydrogel	−20	104 at 0.1 A g^−1^	98% (106)	98% (500) at 0.5 A g^−1^	81 at 0.5 A g^−1^
LiFePO_4_^[^ ^70^ ^]^	Zn	3 mol L^−1^ LiCl + 4 mol L^−1^ ZnCl_2_	−10	92.7 at 0.6C	66.7% (139)	97% (50)	–
FeHCF^[^ ^71^ ^]^	Zn	1 m ZnTFSI + 21 m LiTFSI in PAM hydrogel	−15	39 at 1 A g^−1^	50% (78)	98% (100)	–
Bi_2_S_3_^[^ ^72^ ^]^	Zn	1 m ZnTFSI + 21 m LiTFSI in PAM hydrogel	0	100 at 0.3 A g^−1^	41.7% (240)	–	50 at 6 A g^−1^
V_3_O_7_·H_2_O@ V_2_O_5_·*n*H_2_O^[^ ^17^ ^]^	Zn	21 m LiTFSI + 1 m Zn(CF_3_SO_3_)_2_	−15	60 at 0.5 A g^−1^	27.9% (215)	–	–
Alkaline batteries							
Ni(OH)_2_^[^ ^73^ ^]^	PAQS	10 m KOH	−25	185 at 0.5C	92.5% (200)	–	160 at 10C
NiCo hydroxide^[^ ^74^ ^]^	Zn	6 m KOH + 0.2 m Zn(CH_3_COO)_2_ in PANa hydrogel	−20	126 at 9C	86% (133) at 27C	87% (10 000) at 19C	100 at 36C

## 5 Summary and Outlook

Decent subzero temperature performance is a must for practical application of batteries, in particular a challenge for ARMBs due to the nature of water freeze. While the research on the subzero temperature performance is still in its infancy stage, we noted more and more studies are reported with the blooming of ARMBs. We have summarized strategies and mechanisms adopted to improve subzero temperature performance of ARMBs with an emphasis on freeze behavior of water. Here, we would like to highlight the following issues:

1. In general, the subzero temperature performance of ARMBs is still inferior to their organic electrolyte counterparts due to the fact that the freezing point of water is higher than most organic materials. This can be reflected from the lowest temperature achieved and capacity retention achieved at the same temperature.
2. Studies on improvement electrolyte always get organic materials involved, including additives, hydrogel, or special salts. While it is not unacceptable, to keep the competitiveness of ARMBs, more attention should be paid to maintaining their intrinsic safety and low cost not being sacrificed by the organic materials adopted.
3. Most studies on improving subzero temperature performance of ARMBs are concentrated on electrolyte modification. While electrolyte modification is effective, it should be noted that the electrolyte deterioration is also not avoidable at low temperature. On the other hand, electrode and nonactive materials are also related to subzero temperature performance of ARMBs as they provide transportation channels for ions and electrons. In contrast to nonavoidable performance deterioration of electrolyte, some electrode materials may exhibit even better conductivity at low temperatures, which may provide a unique opportunity for ARMBs working at subzero temperatures.

In recent years, studies on water and hydrogel have achieved remarkable progress. Digging out ideas from these researches may provide unique opportunities to studies on ARMBs working at subzero temperatures.

## Conflict of Interest

The authors declare no conflict of interest.
